# Iatrogenic genitourinary fistula: an 18-year retrospective review of 805 injuries

**DOI:** 10.1007/s00192-014-2445-3

**Published:** 2014-07-26

**Authors:** Thomas J. I. P. Raassen, Carrie J. Ngongo, Marietta M. Mahendeka

**Affiliations:** 1 c/o AMREF Clinical Services, P.O. Box 30125, 00100 Nairobi, Kenya; 2 Fistula Care Plus, EngenderHealth, ABC Place, Rear Entrance, 2nd Floor, Waiyaki Way, P.O. Box 57964-00200, Nairobi, Kenya; 3 Ministry of Health, Mwanza, Tanzania

**Keywords:** Cesarean section, Genitourinary fistula, Hysterectomy, Iatrogenic, Ureteric injury

## Abstract

**Introduction and hypothesis:**

Genitourinary fistula poses a public health challenge in areas where women have inadequate access to quality emergency obstetric care. Fistulas typically develop during prolonged, obstructed labor, but providers can also inadvertently cause a fistula when performing obstetric or gynecological surgery.

**Methods:**

This retrospective study analyzes 805 iatrogenic fistulas from a series of 5,959 women undergoing genitourinary fistula repair in 11 countries between 1994 and 2012. Injuries fall into three categories: ureteric, vault, and vesico-[utero]/-cervico-vaginal. This analysis considers the frequency and characteristics of each type of fistula and the risk factors associated with iatrogenic fistula development.

**Results:**

In this large series, 13.2 % of genitourinary fistula repairs were for injuries caused by provider error. A range of cadres conducted procedures resulting in iatrogenic fistula. Four out of five iatrogenic fistulas developed following surgery for obstetric complications: cesarean section, ruptured uterus repair, or hysterectomy for ruptured uterus. Others developed during gynecological procedures, most commonly hysterectomy. Vesico-[utero]/-cervico-vaginal fistulas were the most common (43.6 %), followed by ureteric injuries (33.9 %) and vault fistulas (22.5 %). One quarter of women with iatrogenic fistulas had previously undergone a laparotomy, nearly always a cesarean section. Among these women, one quarter had undergone more than one previous cesarean section.

**Conclusions:**

Women with previous cesarean sections are at an increased risk of iatrogenic injury. Work environments must be adequate to reduce surgical error. Training must emphasize the importance of optimal surgical techniques, obstetric decision-making, and alternative ways to deliver dead babies. Iatrogenic fistulas should be recognized as a distinct genitourinary fistula category.

## Introduction

A genitourinary fistula is an abnormal communication between the bladder and/or the urethra and the vagina. Most genitourinary fistulas result from prolonged, obstructed labor: the presenting part of the fetus compresses tissues against the pelvic bones, causing pressure necrosis [1]. A hole forms as the tissue dies, typically rendering the woman incontinent. Fistulas are a recurring problem in areas where women have inadequate access to quality emergency obstetric care [2].

Not all genitourinary fistulas are obstetric. Health providers may inadvertently cause injury to the urinary tract during obstetric or gynecological surgery. Other causes of fistulas include carcinoma of the cervix, radiotherapy, and sexual violence [3–5].

An iatrogenic genitourinary fistula (IF) is an abnormal communication between the bladder or ureter and the uterus/cervix/vagina, resulting from a surgical procedure. Any surgery carries some risk of provider error. IFs are typically caused during cesarean section (CS), ruptured uterus repair, hysterectomy for ruptured uterus, and gynecological hysterectomy.

Ureteric injuries, also known as uretero-(cervico)-vaginal fistulas, are nicks, cuts, or ties in the distal ureter where it is nearest to the cervix. A vault fistula is a connection between the bladder and the apex of the vagina (vault), following total abdominal hysterectomy. A vesico-[utero]/-cervico-vaginal fistula (VCVF) is an accidental bladder injury (cut or suture) made during a CS or CS/subtotal hysterectomy that creates a passage between the bladder and the uterus/cervix and vagina. Waaldijk has developed a classification system for genitourinary fistulas based on anatomy and physiology [1]. Vault fistulas and VCVFs are both classified as type I; ureteric injuries as type III.

The objective of this paper is to evaluate the frequency and characteristics of IF types among fistula patients, as well as the risk factors for IF occurrence, in some countries in Africa and Asia.

## Materials and methods

This retrospective record review evaluated the frequency and characteristics of IFs within a series of operations for fistulas carried out by the authors and colleagues in places where women often have inadequate access to quality emergency obstetric care. Data were collected between June 1994 and September 2012 in 65 facilities across 11 countries, mostly in eastern Africa (Table 1). The first author developed a standard form for collecting data on all women undergoing fistula repair surgery. The forms were completed by the surgeon who interviewed the patient and performed the fistula surgery. Data were then entered into an Excel database. No patient names were stored in the electronic database; each woman was instead assigned a unique patient identification number. Approval for this retrospective record review was granted by the AMREF Ethics and Scientific Review Committee. Data were analyzed using Stata software (version 2007; StatCorp, College Station, TX, USA).

**Table 1:** Women with iatrogenic fistula: country of repair

Country	Iatrogenic fistulas	
	*n*	%
Tanzania	286	36.3
Uganda	238	30.2
Kenya	162	20.6
Rwanda	47	6.0
Malawi	22	2.8
South Sudan	9	1.1
Zambia	6	0.8
Ethiopia	6	0.8
Somalia	5	0.6
Bangladesh	4	0.5
Afghanistan	3	0.4
Total	788	

The study is based on records from 5,959 women who underwent fistula repair surgery. Of these, 788 women were identified as having one or more IFs. The first and third authors interviewed all the women before surgery and performed 92 % of the IF surgeries included in this data series. They assisted with or were present for the remainder. In all cases, the operating surgeon noted the classification during the fistula surgery (ureteric, vault, or VCVF).

On the basis of pre-surgery patient interviews, the operating surgeon documented the woman’s age at presentation, age at fistula development, height, duration of leaking, parity, education level, living situation, and profession. The surgeon discussed whether the patient had undergone any previous laparotomies (number and type) or surgery for fistula repair (number and outcome).

The operating surgeon noted which procedure caused the IF, whether obstetric or gynecological, and the interval in days between the causative procedure and the start of leaking. The analysis divided women who developed IF following an obstetric procedure into subgroups: CS; repaired ruptured uterus; and hysterectomy for ruptured uterus (CS/hysterectomy). For obstetric IF patients, the surgeon noted the baby’s sex and whether it was alive or stillborn.

For analysis, ureteric injuries were grouped according to causative surgery: CS, ruptured uterus repair, hysterectomy for ruptured uterus, or gynecological hysterectomy. All vault fistulas were caused during total abdominal hysterectomies; they were grouped according to whether the causative hysterectomy was for obstetric or gynecological indications. VCVFs were divided into those women with a live baby and those with a stillbirth. In cases of multiple births, if at least one baby was living, the mother was counted in the live-baby group. Two women had obstetric fistulas and developed vault fistulas during hysterectomies that attempted to correct urinary leaking. Given that the iatrogenic injuries occurred during their hysterectomies, they were counted in the gynecological hysterectomy group.

Iatrogenic fistulas can be considered to cover a spectrum, ranging from “definitely iatrogenic” to “likely iatrogenic.” Three groups of fistulas are definitely iatrogenic. The location of ureteric injuries indicates accidental injury by a health provider. All ureteric injuries are iatrogenic, whether following CS, CS/hysterectomy, or planned gynecological hysterectomy. Vesico-vaginal vault fistulas appearing after hysterectomy for gynecological reasons, such as fibroids, are iatrogenic. Finally, the delivery of a live baby by CS is rarely associated with pressure necrosis [6]. If the baby is living, VCVF located between the lower segment of the uterus/cervix and the bladder strongly suggests an accidental bladder injury (suture or cut) during a CS.

Vault fistulas following emergency hysterectomy for a ruptured uterus or CS/hysterectomy are probably iatrogenic. A ruptured uterus can involve the bladder as well, in which case the fistula would be obstetric, but the bladder can also be damaged during dissection of the lower uterine segment and cervix, in particular when aggravated by a prior CS: through tearing and/or damaging the blood supply during blunt dissection, or including the bladder in the suture line while closing the vaginal apex.

Vesico-[utero]/-cervico-vaginal fistulas following CS for a stillborn baby are likely to be iatrogenic. In cases where the baby was lost, this analysis included VCVFs less than 3 cm and located clearly in the cervical canal, based on author experience.^1^ Women who had a ruptured uterus and stillborn baby were excluded, given the possibility of a ruptured bladder and therefore an obstetric rather than an iatrogenic cause. A patient history of previous CS or live birth increases the likelihood that the injury is iatrogenic.

The first author noted the cadre of health provider performing the causative procedure, on the basis of the description provided by the woman and his knowledge of local facilities and their staffing, which was complemented by input from the local staff. For this analysis, health providers were grouped as follows: clinical officers and assistant medical officers (CO/AMO); medical officers (MO); registrars; and specialists. CO/AMOs typically do not have a university qualification before completing at least 3 years of medical training and being licensed to provide general medical services. MOs have 5 years of medical training, plus an internship in medicine, pediatrics, surgery, and obstetrics/gynecology. Registrars are residents in a medical specialty; specialists have completed residency training and are qualified in their specialty.

Of the 788 women with one or more IFs, 18 women had two types of IFs concurrently: 9 had their fistulas repaired over multiple surgeries, 8 had both fistulas repaired in one surgery, and 1 woman had only one of her injuries repaired. The frequencies of fistula characteristics according to classification considered the total of 805 IFs repaired by the author and colleagues. For example, if a woman had both a ureteric injury and a vault fistula, she was included as a member of both the ureteric and vault groups. Two women required multiple repair attempts to close a single fistula, with both surgeries performed by the author and colleagues. Only one record from each of these women was included in the analysis, so that the fistulas would not be counted twice. Data are presented according to the strength of the evidence of iatrogenic origin.

## Results

**Fig. 1:**
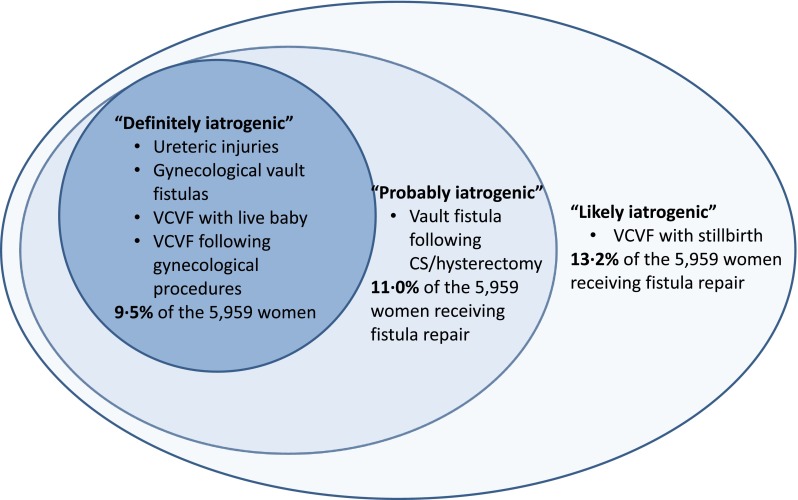
Conceptual categorization of iatrogenic fistulas

The 788 women experiencing IF represent 13.2 % of the 5,959 women in this series. Table 1 presents the breakdown by country. Four-fifths of the women (632, 80.2 %) developed an IF following surgery for obstetric complications (Table 2). The others (156, 19.8 %) developed IF following a gynecological procedure, nearly always hysterectomy. Of the 5,959 women undergoing fistula repair, 9.5 % had a fistula in one of the “definitely iatrogenic” categories outlined in the Materials and methods section. The cumulative percentage of “definitely” or “probably iatrogenic” was 11.0 %, while the cumulative percentage of “definitely,” “likely,” or “probably iatrogenic” was 13.2 % (Fig. 1).

**Table 2:** Procedures in which iatrogenic fistula occurred

Procedure causing iatrogenic fistula	Waaldijk classification						
	Type I (vesico-cervico-vaginal fistula)		Type II (vault fistula)		Type III (ureteric injury)		Total (100 %)
	*n*	%	*n*	%	*n*	%	
Obstetric procedures							
Cesarean section	324	70.1	0	0	138	29.8	462
Repair of ruptured uterus	9	36.0	0	0	16	64.0	25
Hysterectomy for ruptured uterus	16	10.0	86	54.1	57	35.8	159
Gynecological procedures							
Gynecological hysterectomy	1	0.6	95	60.1	62	39.2	158
Other	1	100.0	0	0	0	0	1
Total	351		181		273		805

Women in the obstetric group were generally younger, shorter, and had suffered longer with their fistula compared with those in the gynecological group (Table 3).

**Table 3:** Demographic profile of iatrogenic fistula patients, by causative procedure type

	Obstetric cause (*n* = 632 women)	Gynecological cause (*n* = 156 women)	t	*p* value
	Mean, SD (range)	Mean, SD (range)		
Age at fistula occurrence	28.6 years, 6.9 (13–48)	41.9 years, 8.5 (24–76)	18.17	0.000
Age at time of fistula repair	31.5 years, 8.5 (14–61)	44.0 years, 8.5 (25–76)	16.31	0.000
Height	153.3 cm, 6.4 (128–171)	158.5 cm, 8.0 (115–179)	7.35	0.000
Parity	4.5, 2.7 (1–12) 13.6 % primiparous	4.2, 3.2 (0–15) 9.2 % nulliparous	−0.68	NS
Duration of leaking	36.8 months, 62.2 (1 month to 34 years)	25.2 months, 46.5 (1 month to 26 years)	−2.59	0.010

In addition to iatrogenic injuries, 28 women (3.6 %) had obstetric fistula and 2 women had perineal tears at the time of presentation. Eight women had undergone successful fistula repair in the past, for a previous or concurrent fistula. A total of 45 women (5.7 %) had undergone at least one past unsuccessful attempt at surgery, whether for an iatrogenic or concurrent obstetric fistula.

Of the 805 IFs, 273 (33.9 %) were ureteric injuries, 181 (22.5 %) were vault fistulas, and 351 (43.6 %) were VCVFs (Table 2). Over half of the procedures resulting in IF were CS (462, 57.4 %; Table 2). Hysterectomy was the next most frequent cause, accounting for 317 iatrogenic injuries (39.4 %): 159 hysterectomies were for ruptured uterus (19.8 %) and 158 (19.6 %) were for gynecological indications. Twenty-five women (3.1 %) were injured during repair of a ruptured uterus; 1 was injured during an induced abortion.

Medical officers (MOs) performed over half of the causative procedures (474, 58.9 %; Table 4). Clinical officers/assistant medical officers (CO/AMOs) performed 231 causative procedures overall (28.7 %) and 73.8 % of the 313 procedures in Malawi and Tanzania. Specialists performed 78 (9.7 %), while registrars performed 22 (2.7 %).

**Table 4:** Cadre of staff performing causative procedure, by procedure

Procedure causing iatrogenic fistula	Cadre of staff performing causative procedure								Total (100 %)
	Clinical officer/assistant medical officer		Medical officer		Registrar		Specialist		
	*n*	%	*n*	%	*n*	%	*n*	%	
Obstetric procedures									
Cesarean section	148	32.0	297	64.3	10	2.2	7	1.5	462
Repaired ruptured uterus	10	40.0	14	56.0	1	4.0	0	0	25
Hysterectomy for ruptured uterus	41	25.8	102	64.2	6	3.8	10	6.3	159
Gynecological procedures									
Gynecological hysterectomy	31	19.6	61	38.6	5	3.2	61	38.6	158
Other	1	100	0	0	0	0	0	0	1
Total	231		474		22		78		805

A quarter of the women with IF had undergone previous laparotomy (201, 25.5 %; Table 5). A quarter (24.9 %) of these women had undergone more than one laparotomy. Nearly all laparotomies (197, 98.0 %) were CS; others were bilateral tubal ligation, myomectomy, and salpingectomy.

**Table 5:** Previous laparotomy among women with iatrogenic fistula

	Fistulas	Women	Women with previous laparotomy		Among women with previous laparotomy, number of laparotomies undergone						Type of laparotomy
					1		2		3		
			*n*	%	*n*	%	*n*	%	*n*	%	
Total	805	788^b^	201	25.5	151	75.1	42	20.9	8	4.0	98 % CS
											2 % other
Ureteric injury	273	268	31	11.6	20	64.5	9	29.0	2	6.5	100 % CS
Vault fistula	181	181	31	17.1	24	77.4	4	12.9	3	9.7	87 % CS
											13 % other^c^
VCVF: overall	351^a^	351^a^	141	40.2	108	76.6	30	21.3	3	2.1	100 % CS
Live baby	210	210	102	48.6	76	74.5	23	22.6	3	2.9	
Stillbirth	139	139	39	28.1	32	82.0	7	18.0	0	0	

^a^The two women with vesico-[utero]/-cervico-vaginal fistulas (VCVF) following gynecological procedures are not included in either subgroup

^b^Women with multiple iatrogenic fistulas are counted once in each applicable group

^c^Other laparotomies included bilateral tubal ligation, myomectomy, and salpingectomy

### Ureteric injuries

The 273 ureteric injuries constituted 33.9 % of the 805 IFs considered (Table 2). More than three-quarters (211 out of 273, 77.3 %) of the ureteric injuries occurred following obstetric procedures. Half of the ureteric injuries occurred during CS (138, 50.6 %). Sixteen ureteric injuries (5.9 %) followed a repair of a ruptured uterus, while 57 (20.9 %) presented following a hysterectomy for ruptured uterus. Sixty-two ureteric injuries (22.7 %) followed gynecological hysterectomy.

Medical officers performed over half (145, 53.1 %) of the procedures causing ureteric injury. CO/AMOs performed 93 (34.1 %), specialists 26 (9.5 %), and registrars 9 (3.3 %). The mean interval between the causative procedure and the start of leaking was 10.4 days; the median was 7 days (IQR: 3–14). The left ureter was more likely to be injured than the right: 169 cases (61.9 %) vs 104 cases (38.1 %). Five women had injuries to both ureters. Thirty-one women with ureteric injury had undergone one or more previous CS (Table 5).

### Vault fistulas

The 181 vault fistulas represented 22.5 % of the 805 IFs considered (Table 2). The vault fistulas were all caused during a total abdominal hysterectomy: 95 (52.5 %) for gynecological reasons and 86 (47.5 %) for a ruptured uterus.

Medical officers performed 95 (52.5 %) of the procedures that caused vault fistula; specialists, 48 (26.5 %); CO/AMOs, 30 (16.6 %); registrars, 8 (4.4 %). The mean interval between the causative procedure and the start of leaking was 7.0 days; the median was 3 days (IQR: 0–8).

### Vesico-[utero]/cervico-vaginal fistulas

The 351 VCVFs represent 43.6 % of the 805 IFs (Table 2). Nearly all VCVFs were associated with obstetric procedures (349, 99.4 %), with 210 associated with a live birth and 139 associated with a stillbirth. The remaining two VCVFs followed gynecological procedures (1 subtotal hysterectomy and 1 induced abortion).

Over 40 % of women with VCVF (141) had undergone a previous laparotomy, all CS. Women who gave birth to live babies were more likely to have had a previous CS than women who experienced stillbirth (Table 5). MOs performed 66.7 % of the causative operations (234), CO/AMOs performed 30.8 % (108), registrars 1.4 % (5), and specialists 1.1 % (4). All but one of the VCVFs associated with stillbirth were caused during CS. The mean interval between the causative procedure and the start of leaking was 4.8 days; the median interval was 2 days (IQR: 0–7).

## Discussion

Obstetric fistula is a recognized consequence of health system failure and the inability of the world’s poorest and most marginalized women to access emergency obstetric care [2]. This study considers a sample of women—over 13 % of those presenting for fistula repair surgery—who suffered not directly because of prolonged, obstructed labor, but because of accidents caused by health providers. The prevalence of IF points to gaps in the quality of obstetric and gynecological surgery. The characteristics of iatrogenic injuries prompt us to consider opportunities to improve the quality of service provision.

A range of obstetric and gynecological procedures led to the development of IF, including CS (57.4 %), CS/hysterectomy (19.8 %), gynecological hysterectomy (19.6 %), ruptured uterus repair (3.1 %), and induced abortion (0.1 %). Other surgeries, such as destructive vaginal operations or symphysiotomy, also carry risks of accidental harm from the provider [7], but none was reported to be a causative procedure in this series.

Data on whether the causative CSs were elective or emergency were not collected, but data on the duration of labor suggest that 15 women had an elective CS (labor ≤1 h). The mean duration of labor reported by the remaining women who underwent CS or CS/hysterectomy was 39.7 h. Women experiencing obstetric complications frequently present to the hospital late, and providers may have had inadequate time to prepare their patients [8]. The availability of skilled professionals able to perform surgery is likely to be particularly low outside of normal working hours [8, 9].

The median age of women who developed IF during a gynecological procedure was 42 years, which is consistent with the patient population requiring gynecological procedures and is in line with published data [8, 10, 11]. The median age of women who developed IF during an obstetric procedure was 28 years, older than the age reported for most obstetric fistula patients [3, 5, 12, 14].

Several factors are suspected to place a woman at risk of IF. These include prior uterine operation, endometriosis, cervical myoma, and prior pelvic radiation [10, 11, 14]. Scar tissue and adhesions from prior laparotomies can create challenges for providers performing obstetric and gynecological surgery. It is therefore reasonable to hypothesize that obstetric or gynecological surgery might carry a greater risk of iatrogenic injury for women who have undergone a laparotomy in the past [10]. The frequency of previous laparotomy in the general population is unknown, but in this sample of women with IF, a full quarter had undergone one or more previous laparotomies. One quarter of the women who had had a previous laparotomy had undergone more than one. In this series, 98.0 % of previous laparotomies were cesarean sections.

The different types of IF were not equally associated with previous laparotomy. Under 12 % of ureteric injuries and 17.1 % of vault fistulas occurred in women who had undergone previous laparotomy, but 40.2 % of women with VCVF had undergone at least one previous laparotomy. Among VCVF patients, previous CS was more common in women who delivered a live baby than in those with a stillbirth (48.6 % vs 28.1 %). Providers are more prompt in providing CS to women with a previous CS, and more often as an elective procedure.

While appropriate cesarean sections improve maternal and perinatal outcomes, they do not confer similar advantages when performed in low-risk groups [15]. The World Health Organization has pointed out the intrinsic risk associated with CS [16]; yet, obstetric practice is shifting from vaginal to cesarean birth in many parts of the world, including in some of the countries included in this study [15–18]. It would seem that one risk associated with CS, particularly repeated CS, is that providers might be more likely to accidentally cause iatrogenic injury during a subsequent surgery.

Early detection of IF can help patients avoid prolonged morbidity and its consequences. Early management of IFs should be feasible, so long as providers recognize the problem [8, 14, 19]. Providers can identify many IFs when removing the Foley catheter shortly after surgery. Excluding ureteric injuries, a substantial number of small IFs could be healed by re-introducing the catheter and leaving it for a period of 4–6 weeks, with a regimen of plenty of oral fluids and sitz baths [20].

The median time before patients began leaking among those with a VCVF or vault fistula was 2 and 3 days respectively, and 7 days after the causative surgery in those with ureteric injury. In this analysis, IFs following gynecological hysterectomy were treated earlier than those following obstetric surgeries. Differences in patient populations may explain this finding: gynecological patients are mainly self-referred, establishing a relationship with a provider who may recognize the problem and ensure appropriate care. If the leaking starts after discharge, the patient will go back to the operating provider and will be referred appropriately. Obstetric patients typically arrive as emergencies, and the operating provider may not see the patient after her CS. Moreover, leaking after an emergency CS could be due to pressure necrosis; providers may not immediately recognize the iatrogenic cause.

It appears that the ureters are not at equal risk of being accidentally damaged during CS [8, 21]. The left ureter is more likely to be affected during CS for several reasons. First, it is half a centimeter nearer to the cervix than the right ureter [22]. Second, the large sigmoid colon in African women causes dextro-rotation of the gravid uterus, bringing the left ureter forward [23]. Finally, many right-handed operators stand on the right side of the patient when performing CS, making it more likely to inadvertently injure the left ureter.

All cadres of health providers in this series performed procedures that resulted in IF, from assistant medical officers to specialists. National data on the cadres of staff performing different types of procedures are typically unavailable, but the profile described here is not surprising based on the human resources in the countries involved. Medical officers are typically the most likely cadre to carry out emergency surgeries such as CS or CS/hysterectomies; thus, their role in 59 % of IFs likely reflects their high involvement in at-risk procedures.

In both Malawi and Tanzania, nonphysician clinicians perform the majority of obstetric surgery. In Malawi, 88 % of emergency obstetric operations in district hospitals are performed by clinical officers [24]. In one study in Tanzania, more than 85 % of obstetric and gynecological surgeries were performed by assistant medical officers [25]. In light of this, the 73.8 % of IFs caused by CO/AMOs in Malawi and Tanzania seems reasonable. In all countries, specialists would be more likely to conduct elective procedures than emergency ones. This explains why specialists performed only 9.6 % of the causative procedures, but 38.6 % of the gynecological hysterectomies that resulted in IF.

This analysis has several limitations. First, the women themselves provided much of the information recorded in the patient records. While women mostly know their obstetric histories, recall can be a challenge. In some cases, providers may not have fully educated patients about procedures performed and the reasons for performing them. The fistula surgeon’s assessment complemented each woman’s account, helping to determine the most likely obstetric history. The first author’s determination of the cadre causing IF was dependent on his and his colleagues’ knowledge of local facilities and their staffing, which could be subject to recall bias and may be difficult for others to reproduce. Finally, these data do not indicate the overall prevalence of IF, but instead point to the proportion of fistula of iatrogenic origin among women in need of fistula repair surgery. This series of IF repairs was drawn from a sample of nearly 6,000 fistula repairs that took place in 65 facilities across 11 countries, ranging from sub-district hospitals to tertiary referral facilities. Reliable population-level information about fistula prevalence is unavailable, as is information about the total number of obstetric and gynecological surgical procedures performed. This means that some of the denominators that would put the findings into context are unavailable.

Prevention of IF is an urgent matter that needs to be addressed in developing countries. Providers performing obstetric and gynecological surgery must have the appropriate competencies. Training, combined with mentoring and ongoing supervision, is essential. In addition, women with obstructed labor must be able to quickly access a health facility with the staffing and infrastructure to provide high-quality emergency obstetric care. As such, it is critical to strengthen referral systems (emergency communication and transport) and address financial barriers that result in delays in care-seeking behavior. Training and equitable deployment of skilled birth attendants at all levels of the health system will ensure that providers can recognize signs of abnormal labor progression and make appropriate decisions about referral. Such efforts should be complemented by community-level interventions that promote household preparation for birth, increase male partner involvement in maternal health, and empower women to take action to ensure their own health and well-being.

Whenever women arrive at facilities in need of care, it is critical that providers are able to make informed and timely decisions. Facilities and providers must consider the quality of emergency obstetric care, including the decision-making process leading to a CS. The partograph is an essential tool for monitoring progress in labor and for diagnosing obstructed labor [26]. Providers must manage women in labor according to best medical practice; some women wait hours or days in a hospital before receiving any intervention [27, 28]. Facilities must likewise treat providers fairly, compensating them for their service and providing adequate resources, training, and supervision.

Providers must have the knowledge and experience to be able to provide high-quality services. As shown elsewhere [6, 7], the high number of cesarean deliveries for dead infants emphasizes the importance of re-evaluating alternative vaginal delivery methods. Many providers may benefit from training on alternatives to cesarean section, including vacuum extraction, symphysiotomy, and craniotomy for dead babies. Provider training should likewise highlight optimal operative techniques [9]. In dissection of the bladder and the lower uterine segment, blunt dissection can damage blood supply to the bladder, especially if the patient has had a previous CS. Sharp dissection is always preferable [14, 22]. The training, mentoring, and supervision of providers will improve care and therefore the health outcomes of women.

## Footnotes

1 In cases where the operating surgeon noted a range in estimated size (“1–2,” “2–3”), we conservatively recorded the larger number. A cut-off of 2.5 cm would have included 97.8 % of the sample (136 out of 139). A cut-off of 2 cm would have included 92.1 % (128 out of 139); a cut-off of 1.5 cm would have included 71.2 % (99 out of 139); and a cut-off of 1 cm would have included 52.5 % (73 out of 139).
